# Supplementary material for: Evocalcet with vitamin D receptor activator treatment for secondary hyperparathyroidism
Source: PLoS One. 2022 Feb 17;17(2):e0262829. doi: 10.1371/journal.pone.0262829 (PMC8853539; doi:10.1371/journal.pone.0262829)
Supplement: S1 Table — (DOCX) [file pone.0262829.s001.docx]

**S1 Table.** **List of Institutional Review Boards (IRBs).**

| **Medical Institutions** | **Name of Institutional Review Board** |
| --- | --- |
| Asahikawa-Kosei General Hospital | Asahikawa-Kosei General Hospital IRB |
| Nikko Memorial Hospital | Nikko Memorial Hospital IRB |
| Sapporo Hokuyu Hospital | Sapporo Medical Association's IRB |
| Higashi-Naebo Hospital | Sapporo Medical Association's IRB |
| H • N • MEDIC | Sapporo Medical Association's IRB |
| H • N • MEDIC Kita-Hiroshima | Sapporo Medical Association's IRB |
| H • N • MEDIC Sapporo Higashi | Sapporo Medical Association's IRB |
| Kojinkai Chuou Clinic | Koyasu Neurosurgical Clinic IRB |
| Kimachi Hospital | Koyasu Neurosurgical Clinic IRB |
| Rifunonaika Clinic (Miyata Rifu Clinic) | Koyasu Neurosurgical Clinic IRB |
| Dainohara Clinic | Koyasu Neurosurgical Clinic IRB |
| Kawadaira Naika Medical Clinic | Koyasu Neurosurgical Clinic IRB |
| Tokiwakai Joban Hospital | Koyasu Neurosurgical Clinic IRB |
| Ibaraki Seinan Medical Center Hospital | Review Board of Human Rights and Ethics for Clinical Studies |
| Tokiwa Clinic | Koyasu Neurosurgical Clinic IRB |
| Kikuchi Medical Clinic | Koyasu Neurosurgical Clinic IRB |
| Ohishi Naika Clinic | Tokyo Midtown Clinic IRB |
| Tsuchiura Beryl Clinic | Koyasu Neurosurgical Clinic IRB |
| Ora Hospital | Review Board of Human Rights and Ethics for Clinical Studies |
| Heisei Hidaka Clinic | Review Board of Human Rights and Ethics for Clinical Studies |
| Kubojima Clinic | Koukeikai Sugiura Clinic IRB |
| Bosei Hospital | Medical Corporation SHOWAKAI IRB |
| Hakuyukai Yuai Nisshin Clinic | Hoshikuma Dermatology / Allergy Department Clinic IRB |
| Oshima Clinic | Koukeikai Sugiura Clinic IRB |
| Saitama Tsuki no Mori Clinic | Kojinkai Medical Corporation Shin-Nihonbashi Ishii Clinic IRB |
| Japan Community Health Care Organization Chiba Hospital | Eijyukai Ryohoku Hospital IRB |
| Asahi General Hospital | Asahi General Hospital IRB |
| Kohitsujikai Naganuma Clinic | Review Board of Human Rights and Ethics for Clinical Studies |
| Tokatsu Clinic Mirai | Review Board of Human Rights and Ethics for Clinical Studies |
| St. Luke’s International Hospital | St.Luke's International Hospital IRB |
| Toranomon Hospital | Toranomon Hospital and Toranomon Hospital Kajigaya IRB |
| Shinshikai Hachioji Azumacho Clinic | Koukeikai Sugiura Clinic IRB |
| Ayase Ekimae Jin Clinic | Medical Corporation SHOWAKAI IRB |
| Futago-tamagawa Ekimae Clinic | Koukeikai Sugiura Clinic IRB |
| Showakai Bosei Shinjuku Minamiguchi Clinic | Medical Corporation SHOWAKAI IRB |
| Kita Hachiouji Clinic | Medical Corporation SHOWAKAI IRB |
| Toshiba Rinkan Hospital | Koyasu Neurosurgical Clinic IRB |
| Bosei Fujisawa Clinic | Medical Corporation SHOWAKAI IRB |
| Shinjinkai Yokosuka Clinic | Kojinkai Medical Corporation Shin-Nihonbashi Ishii Clinic IRB |
| Maeda Institute of Renal Research Musashikosugi Clinic | Koyasu Neurosurgical Clinic IRB |
| Tachibanadai Hospital | Koukeikai Sugiura Clinic IRB |
| Sekishinkai Kawasaki Clinic | Koukeikai Sugiura Clinic IRB |
| Bosei Hiratsuka Clinic, Medical Corporation Showakai | Medical Corporation SHOWAKAI IRB |
| Boseikai Honatsugi Medical Clinic | Medical Corporation SHOWAKAI IRB |
| Koshikawakinen Yokohama Jin Clinic | Review Board of Human Rights and Ethics for Clinical Studies |
| Shinjinkai Kurihama Clinic | Kojinkai Medical Corporation Shin-Nihonbashi Ishii Clinic IRB |
| Maeda Institute of Renal Research Shin-Yokohama Clinic | Koyasu Neurosurgical Clinic IRB |
| Niigata City Shakai Jigyo Kyokai Shinrakuen Hospital | Niigata City Shakai Jigyo Kyokai Shinrakuen Hospital IRB |
| Fujikoshi Hospital | Kojinkai Medical Corporation Shin-Nihonbashi Ishii Clinic IRB |
| Ueda Jinzo Clinic | Review Board of Human Rights and Ethics for Clinical Studies |
| Japanese Red Cross Society Suwa Hospital | Review Board of Human Rights and Ethics for Clinical Studies |
| Aizawa Hospital | Aizawa Hospital IRB |
| Ueda Tohseki Clinic | Review Board of Human Rights and Ethics for Clinical Studies |
| Kanno Dialysis & Vascular Access Clinic | Review Board of Human Rights and Ethics for Clinical Studies |
| Miyaji Clinic | Kojinkai Medical Corporation Shin-Nihonbashi Ishii Clinic IRB |
| Fuji Daiichi Clinic | Medical Corporation SHOWAKAI IRB |
| Fuyo Association Seirei Numazu | Fuyo Association Seirei Numazu IRB |
| Masuko Clinic Subaru | Masuko Memorial Hospital IRB |
| Masuko Hospital | Masuko Memorial Hospital IRB |
| Daiyukai Daiichi Hospital | Daiyukai Daiichi Hospital IRB |
| Meiyo Clinic | Review Board of Human Rights and Ethics for Clinical Studies |
| Sanen Medi Mates Toyohashi Mates Clinic | Sanen Medi Mates Toyohashi Mates Clinic Sleep Center IRB |
| Anjyo Kyoritsu Clinic | Kaikoukai Healthcare Corporation Nagoya Kyoritsu Hospital IRB |
| Takeuchi Hospital | Kondo Hospital IRB |
| Nishijin Hospital | Koyasu Neurosurgical Clinic IRB |
| Tojinkai Hospital | Kojinkai Medical Corporation Shin-Nihonbashi Ishii Clinic IRB |
| Rakuwakai Otowa Memorial Hospital | Rakuwakai Otowa Hospital IRB |
| Kyotoekimae Takeda Tohseki Clinic | Koseikai Takeda Hospital Group IRB |
| Fuchu Hospital | Koukeikai Sugiura Clinic IRB |
| Soryukai Inoue Hospital | Soryukai Inoue Hospital IRB |
| General Hospital Higashikouri | Review Board of Human Rights and Ethics for Clinical Studies |
| Nozaki Clinic | Koukeikai Sugiura Clinic IRB |
| Shirasagi Minami Hospital | Review Board of Human Rights and Ethics for Clinical Studies |
| Chibune Kidney and Dialysis Clinic, Aijinkai Chibune General Hospital | Aijinkai Chibune General Hospital IRB |
| Shigei Medical Research Hospital | Shigei Medical Research Hospital IRB |
| Sowakai Shigei Hospital | Kojinkai Medical Corporation Shin-Nihonbashi Ishii Clinic IRB |
| Chuo Naika Clinic | Koyasu Neurosurgical Clinic IRB |
| Ichiyokai Clinic | Koukeikai Sugiura Clinic IRB |
| Nakajima Tsujiya Clinic | Akane Foundation Tsuchiya General Hospital IRB |
| Saint Hill Hospital | Koukeikai Sugiura Clinic IRB |
| Tokuyama Central Hospital | Tokuyama Central Hospital IRB |
| Kawashima Tohseki Clinic | Social medical corporation Kawashima society Kawashima Hospital IRB |
| Kinashi Obayashi Hospital | Koukeikai Sugiura Clinic IRB |
| Takayama Hospital | Hoshikuma Hifuka Allergy Clinic IRB |
| Shin Koga Hospital | Shin Koga Hospital IRB |
| Nagasaki Jin Hospital | Tokyo Midtown Clinic IRB |
| Matsushitakai Akebono Clinic | Kojinkai Medical Corporation Shin-Nihonbashi Ishii Clinic IRB |
| Seijinkai Ikeda Hospital | Seijinkai Ikeda Hospital IRB |
| Tomishiro Central Hospital | Koyasu Neurosurgical Clinic IRB |
