# Supplementary material for: Evocalcet with vitamin D receptor activator treatment for secondary hyperparathyroidism
Source: PLoS One. 2022 Feb 17;17(2):e0262829. doi: 10.1371/journal.pone.0262829 (PMC8853539; doi:10.1371/journal.pone.0262829)
Supplement: S2 Table — (DOCX) [file pone.0262829.s002.docx]

**S2 Table.** **Standard deviations of the mean levels and mean percent changes from baseline in intact PTH, corrected calcium, and phosphorus levels stratified by concomitant baseline IV VDRA weekly dose in patients with SHPT treated with evocalcet for 30 weeks.**

|  | Weekly VDRA dose | | | Weekly VDRA dose | | |
| --- | --- | --- | --- | --- | --- | --- |
|  | 0 | < 1.5 µg | ≥ 1.5 µg | 0 | < 1.5 µg | ≥ 1.5 µg |
|  | Intact PTH | | | | | |
| Week | SD of mean level (pg/mL) | | | SD of mean percent change (%) | | |
| 0 | 197.7 | 125.8 | 168.0 | 0.0 | 0.0 | 0.0 |
| 1 | 213.7 | 151.2 | 150.2 | 21.9 | 25.1 | 20.8 |
| 2 | 183.8 | 142.2 | 177.0 | 22.2 | 21.8 | 26.6 |
| 3 | 222.3 | 129.6 | 158.7 | 22.0 | 26.6 | 23.1 |
| 4 | 210.6 | 143.4 | 154.5 | 24.6 | 25.8 | 26.3 |
| 5 | 228.5 | 138.4 | 166.0 | 24.6 | 27.8 | 27.2 |
| 6 | 208.0 | 124.9 | 150.6 | 27.5 | 25.2 | 27.2 |
| 7 | 203.1 | 134.3 | 154.4 | 26.2 | 29.6 | 27.7 |
| 8 | 215.4 | 122.3 | 170.0 | 29.8 | 25.7 | 27.7 |
| 9 | 258.5 | 113.2 | 156.5 | 31.5 | 25.3 | 27.5 |
| 10 | 236.3 | 97.1 | 147.9 | 30.5 | 22.5 | 29.2 |
| 11 | 203.3 | 107.3 | 172.8 | 26.2 | 23.3 | 27.3 |
| 12 | 206.8 | 105.9 | 166.9 | 27.0 | 24.2 | 29.3 |
| 13 | 222.1 | 106.3 | 151.4 | 25.5 | 21.5 | 27.4 |
| 14 | 250.7 | 107.1 | 140.9 | 26.7 | 23.8 | 29.3 |
| 15 | 210.6 | 101.5 | 133.2 | 24.9 | 28.2 | 24.6 |
| 16 | 295.3 | 99.2 | 141.8 | 29.4 | 23.0 | 26.1 |
| 17 | 187.1 | 108.5 | 147.1 | 25.3 | 26.5 | 26.9 |
| 18 | 186.1 | 118.3 | 152.5 | 26.8 | 27.5 | 28.8 |
| 19 | 189.4 | 108.7 | 150.5 | 27.7 | 25.4 | 29.2 |
| 20 | 169.1 | 109.0 | 131.8 | 27.5 | 24.3 | 26.0 |
| 21 | 165.8 | 106.4 | 159.5 | 30.2 | 30.1 | 29.8 |
| 22 | 163.1 | 143.3 | 143.4 | 23.7 | 29.0 | 24.0 |
| 23 | 168.9 | 133.2 | 145.0 | 27.3 | 26.1 | 31.2 |
| 24 | 157.4 | 115.4 | 135.7 | 26.6 | 27.5 | 30.1 |
| 25 | 163.0 | 119.2 | 150.4 | 26.9 | 29.1 | 29.3 |
| 26 | 183.7 | 124.6 | 149.1 | 23.5 | 33.3 | 29.9 |
| 27 | 214.9 | 131.6 | 127.2 | 26.9 | 30.1 | 30.5 |
| 28 | 168.1 | 176.0 | 126.6 | 26.0 | 34.4 | 30.9 |
| 29 | 162.9 | 145.8 | 124.6 | 23.5 | 28.2 | 28.4 |
| 30 | 190.1 | 138.8 | 153.4 | 25.3 | 28.6 | 33.5 |
|  | Corrected calcium | | | | | |
| Week | SD of mean level (mg/dL) | | | SD of mean percent change (%) | | |
| 0 | 0.6 | 0.5 | 0.5 | 0.0 | 0.0 | 0.0 |
| 1 | 0.6 | 0.6 | 0.5 | 4.5 | 4.1 | 3.8 |
| 2 | 0.6 | 0.6 | 0.5 | 4.7 | 5.0 | 3.9 |
| 3 | 0.6 | 0.6 | 0.5 | 4.9 | 4.9 | 4.2 |
| 4 | 0.6 | 0.6 | 0.5 | 5.2 | 5.7 | 5.0 |
| 5 | 0.5 | 0.6 | 0.5 | 5.2 | 4.9 | 4.7 |
| 6 | 0.6 | 0.6 | 0.6 | 5.5 | 5.3 | 4.9 |
| 7 | 0.6 | 0.7 | 0.5 | 6.2 | 6.6 | 5.3 |
| 8 | 0.6 | 0.6 | 0.5 | 6.3 | 5.9 | 5.1 |
| 9 | 0.6 | 0.6 | 0.6 | 6.0 | 5.8 | 5.7 |
| 10 | 0.6 | 0.6 | 0.5 | 6.3 | 6.5 | 5.7 |
| 11 | 0.6 | 0.6 | 0.6 | 6.6 | 7.2 | 5.7 |
| 12 | 0.5 | 0.6 | 0.5 | 6.5 | 6.5 | 5.3 |
| 13 | 0.5 | 0.6 | 0.7 | 6.4 | 6.0 | 7.9 |
| 14 | 0.6 | 0.6 | 0.7 | 7.3 | 6.7 | 6.6 |
| 15 | 0.7 | 0.6 | 0.6 | 7.6 | 5.9 | 6.1 |
| 16 | 0.7 | 0.6 | 0.6 | 8.0 | 6.1 | 6.6 |
| 17 | 0.6 | 0.6 | 0.7 | 7.4 | 6.4 | 7.0 |
| 18 | 0.7 | 0.6 | 0.6 | 8.0 | 5.2 | 6.6 |
| 19 | 0.7 | 0.5 | 0.6 | 7.8 | 6.1 | 6.6 |
| 20 | 0.7 | 0.5 | 0.7 | 8.1 | 6.2 | 6.8 |
| 21 | 0.6 | 0.6 | 0.7 | 7.6 | 6.6 | 7.2 |
| 22 | 0.6 | 0.6 | 0.7 | 7.5 | 6.7 | 7.3 |
| 23 | 0.7 | 0.6 | 1.0 | 7.8 | 7.0 | 11.0 |
| 24 | 0.6 | 0.6 | 0.6 | 7.9 | 7.4 | 7.4 |
| 25 | 0.7 | 0.6 | 0.6 | 8.4 | 7.5 | 6.8 |
| 26 | 0.6 | 0.7 | 0.7 | 7.0 | 7.7 | 7.6 |
| 27 | 0.6 | 0.6 | 0.6 | 7.4 | 7.6 | 6.8 |
| 28 | 0.7 | 0.5 | 0.9 | 8.2 | 6.3 | 9.1 |
| 29 | 0.7 | 0.7 | 0.6 | 7.9 | 7.8 | 7.1 |
| 30 | 0.7 | 0.7 | 0.6 | 8.4 | 8.9 | 7.1 |
|  | Phosphorus | | | | | |
| Week | SD of mean level (mg/dL) | | | SD of mean percent change (%) | | |
| 0 | 1.2 | 1.4 | 1.4 | 0 | 0 | 0 |
| 1 | 1.0 | 1.4 | 1.3 | 0 | 0 | 0 |
| 2 | 1.1 | 1.5 | 1.2 | 17.0 | 15.5 | 15.3 |
| 3 | 1.3 | 1.5 | 1.4 | 15.7 | 13.6 | 16.5 |
| 4 | 1.1 | 1.2 | 1.3 | 19.0 | 15.9 | 17.9 |
| 5 | 1.1 | 1.2 | 1.2 | 18.6 | 18.0 | 21.1 |
| 6 | 1.1 | 1.3 | 1.3 | 24.4 | 18.3 | 19.6 |
| 7 | 1.2 | 1.2 | 1.2 | 22.5 | 20.6 | 18.4 |
| 8 | 1.2 | 1.3 | 1.4 | 23.4 | 21.6 | 20.3 |
| 9 | 1.1 | 1.2 | 1.4 | 24.7 | 20.5 | 22.5 |
| 10 | 1.2 | 1.1 | 1.3 | 22.9 | 18.1 | 22.8 |
| 11 | 1.2 | 1.2 | 1.2 | 25.2 | 20.1 | 21.4 |
| 12 | 1.2 | 1.1 | 1.4 | 26.0 | 19.5 | 23.6 |
| 13 | 1.2 | 1.2 | 1.3 | 25.6 | 16.0 | 22.7 |
| 14 | 1.4 | 1.2 | 1.5 | 26.4 | 16.8 | 22.2 |
| 15 | 1.2 | 1.2 | 1.3 | 29.2 | 18.4 | 25.5 |
| 16 | 1.2 | 1.1 | 1.5 | 28.0 | 18.6 | 22.0 |
| 17 | 1.2 | 1.2 | 1.3 | 29.5 | 17.4 | 24.0 |
| 18 | 1.2 | 1.1 | 1.2 | 26.7 | 17.5 | 22.4 |
| 19 | 1.1 | 1.2 | 1.3 | 29.4 | 18.0 | 21.4 |
| 20 | 1.1 | 1.2 | 1.3 | 25.5 | 19.3 | 23.4 |
| 21 | 1.1 | 1.1 | 1.4 | 24.6 | 17.9 | 23.5 |
| 22 | 1.2 | 1.4 | 1.3 | 26.4 | 17.4 | 22.5 |
| 23 | 1.2 | 1.4 | 1.5 | 28.2 | 16.8 | 22.8 |
| 24 | 1.4 | 1.2 | 1.6 | 27.0 | 19.1 | 22.7 |
| 25 | 1.2 | 1.3 | 1.4 | 29.9 | 17.4 | 25.2 |
| 26 | 1.2 | 1.2 | 1.5 | 24.3 | 19.2 | 25.0 |
| 27 | 1.2 | 1.3 | 1.4 | 26.3 | 17.1 | 25.5 |
| 28 | 1.2 | 1.5 | 1.5 | 27.7 | 22.8 | 24.6 |
| 29 | 1.2 | 1.5 | 1.4 | 25.7 | 18.6 | 25.1 |
| 30 | 1.3 | 1.3 | 1.5 | 25.0 | 18.8 | 25.8 |

IV, intravenous; PTH, parathyroid hormone; SD, standard deviation; SHPT, secondary hyperparathyroidism; VDRA, vitamin D receptor activator.
