# Supplementary material for: Evocalcet with vitamin D receptor activator treatment for secondary hyperparathyroidism
Source: PLoS One. 2022 Feb 17;17(2):e0262829. doi: 10.1371/journal.pone.0262829 (PMC8853539; doi:10.1371/journal.pone.0262829)
Supplement: Attachment — Submitted filename: Evocalcet head to head comparison with Cinacalcet .docx [file pone.0262829.s003.docx]

**EVOCALCET PRELIMINARY ANALYSIS**

**Observational studies suggest that in hemodialysis patients the use of a vitamin D receptor (VDR) activator, such as calcitriol, doxercalciferol, paricalcitol, or alfacalcidol,**

This study is an ad hoc analysis of a previous phase 3 head-to-head comparison study of hemodialysis patients with hyperparathyroidism, after 30-day treatment with evocalcet. This study was conducted at 89 study sites in Japan (Oct 2015-Nov 2016) and demonstrated a **non-inferiority to cinacalcet** to suppress iPTH **with fewer gastro-intestinal adverse events**.

Patients were treated according to 3 regimens of IV VDRA dose: no dose group (NDG) , low dose group (LDG < 1.5 ug/week) and high dose group (HDG, >=1.5 ug/week

The objective of this ad hoc analysis was to evaluate the safety and efficacy of combined evocalcet and vitamin D receptor activator on intact parathyroid hormone, corrected calcium, and fibroblast growth factor-23 levels.

**Main results were:**

Intact parathyroid hormone, corrected calcium, phosphorus, and fibroblast growth factor-23 levels decreased in all groups;

phosphorus and fibroblast growth factor-23 levels remained high in the high dose group???

In low and high dose groups, hypocalcemia was less common than in the no dose group (p = 0.014)

~~In low and high dose groups, greater proportions of patients achieved the corrected calcium target, compared with the no dose group (p=0.043)~~

~~Ratios of intact-to-C-terminal FGF-23 decreased in all groups.~~

Evocalcet with concomitant vitamin D receptor activator demonstrated clinical benefits: more patients achieved the corrected calcium target and exhibited decreased fibroblast growth factor-23 synthesis; the incidence of hypocalcemia also decreased and fewer gastrointestinal symptoms were verified.

**Evocalcet head to head comparison with Cinacalcet**

This study is an ad hoc analysis of a previous phase 3, head-to-head comparison study of hemodialysis patients with hyperparathyroidism, after 30-day treatment with evocalcet. This study was conducted at 89 study sites in Japan (Oct 2015-Nov 2016) and demonstrated a non-inferiority to cinacalcet to suppress iPTH with fewer gastro-intestinal adverse events.

This study has demonstrated that the association of oral evocalcet with vitamin D activator was beneficial, with lower synthesis of FGF-23, lower incidence of hypocalcemia and with more patients achieving the calcium target. This oral formulation of evocalcet, was also associated with a reduction of gastric symptoms when compared with oral cinacalcet.

This study confirms the benefit of the treatment of hyperparathyroidism with an association of a calcimimetic + vitamin D.

The reduction of gastric symptoms verified with evocalcet will contribute for a better adherence of this new oral treatment for hyperparathyroidism.

However, the intravenous administration of etelcalcetide after hemodialysis, assures the patient adherence to this kind of treatment.

Evocalcet will be very useful for the treatment of patients with hyperparathyroidism who develop gastric symptoms associated with cinacalcet and who have no conditions for a three times/week intravenous treatment. This will be the case, for instance, of DP patients.
