# Supplementary material for: Evocalcet with vitamin D receptor activator treatment for secondary hyperparathyroidism
Source: PLoS One. 2022 Feb 17;17(2):e0262829. doi: 10.1371/journal.pone.0262829 (PMC8853539; doi:10.1371/journal.pone.0262829)
Supplement: Attachment — Submitted filename: Response to Reviewers.docx [file pone.0262829.s004.docx]

We appreciate the constructive review comments. Below you will find our point-by-point responses to all the reviewers’ comments.

Journal Requirements:

*1. Please ensure that your manuscript meets PLOS ONE's style requirements, including those for file naming.*

**Response:** File names have been corrected accordingly.

*2. We note that you have included the phrase “data not shown” in your manuscript. Unfortunately, this does not meet our data sharing requirements. PLOS does not permit references to inaccessible data. We require that authors provide all relevant data within the paper, Supporting Information files, or in an acceptable, public repository. Please add a citation to support this phrase or upload the data that corresponds with these findings to a stable repository (such as Figshare or Dryad) and provide and URLs, DOIs, or accession numbers that may be used to access these data. Or, if the data are not a core part of the research being presented in your study, we ask that you remove the phrase that refers to these data.*

**Response:** These data are now included as Fig. 3.

*3. Thank you for stating the following in the Competing Interests section: 'TS received consulting fees from Kyowa Kirin Co., Ltd. (KKC), Ono Pharmaceutical, Taisho Toyama Pharmaceutical, Fuji Pharma, and FUSO, and lecture fees from KKC, Chugai Pharmaceutical, Bayer, Kissei Pharmaceutical, Torii Pharmaceutical, Ono Pharmaceutical, and FUSO. SA, YE, and TK are employees of KKC. MF received consulting fees from KKC and Ono Pharmaceutical; lecture fees from KKC, Bayer, Torii Pharmaceutical, and Ono Pharmaceutical; and grants from KKC and Bayer. TA received consulting fees from KKC, Astellas Pharma, Bayer, Fuso Pharmaceutical, Japan Tobacco, Ono Pharmaceutical, Sanwa Chemical, Otsuka, GSK, and NIPRO, and lecture fees from KKC, Chugai Pharmaceutical, Bayer, Kissei Pharmaceutical, Torii Pharmaceutical, and Ono Pharmaceutical. The authors report no other conflicts of interest in this work.'*

*Please confirm that this does not alter your adherence to all PLOS ONE policies on sharing data and materials, by including the following statement: "This does not alter our adherence to PLOS ONE policies on sharing data and materials.” (as detailed online in our guide for authors http://journals.plos.org/plosone/s/competing-interests). If there are restrictions on sharing of data and/or materials, please state these. Please note that we cannot proceed with consideration of your article until this information has been declared.*

**Response:** As instructed, the updated Competing Interests Statement has been included in the cover letter.

**Reviewer #1:** Major comments

*1. If I understand correctly the present analysis has been performed only in the patients of the evocalcet group of the parent head-to-head comparison study. This should be made clear from the outset. In the parent trial 320 patients were randomized to receive evocalcet. In the present ad hoc analysis only 253 patients were included. Why were the remaining 67 patients excluded ?*

**Response:** In the previous phase 3 head-to-head comparison study [Fukagawa et al Kidney Int. 2018;94: 818–825] and this study (Line 149), the efficacy of evocalcet was evaluated in the per protocol set as primary analysis, which was defined as the population of patients included in the full analysis set, excluding the following: those who failed to meet any of the inclusion criteria, or patients who met any of the exclusion criteria; those with a drug compliance of <70% (based on patient reports to investigators at hospital visits); those who received a prohibited concomitant medication or therapy; those with missing iPTH levels at more than one of three time points in the evaluation period; and those with a protocol deviation that may have affected the efficacy evaluation. According to the previous phase 3 head-to-head comparison study, three patients discontinued the study before initiating the treatment and, therefore, 317 patients started the treatment with evocalcet. Of these, 253 were included in the per-protocol set. This information was included in the original article of the phase 3 head-to-head comparison study. To clarify the data source, the citation has been added in Line 177.

*2. VDRA stands for calcitriol and a group of other active vitamin D compounds. Authors further need to tell from the outset which active vitamin D compounds were administered, and to which extent the administered doses were comparable.*

**Response:** Specific active vitamin D compounds have been included in Line 112. A conversion method to show how their weekly doses were estimated has been included in Line 128.

*3. Eighty-one patients among the 117 weekly zero VDRA dose patients were on oral VDRA therapy at baseline. Did they continue or was this therapy stopped at trial initiation ?*

**Response:** As stated in Line 110, initiation of or changes in preparation, dose, and dosing regimen of VDRA medications or derivatives were not permitted from 2 weeks before screening until the end of the study in all patients, meaning that patients in the zero VDRA group continued the same oral VDRA therapy. The sentence in Line 111 has been changed to clarify that the above point applies to the IV and oral VDRA therapies.

*4. In the Results section authors repeatedly mention « trends » for changes of various biochemistry values but actually fail to provide P values in the Figures or Figure legends. Therefore, I conclude that none of these « trends » corresponded to changes reaching the level of statistical significance. The terms « trend » and « trended » should be deleted and instead any non significant change should be acknowledged as no change. This will require a serrious revision of the interpretation of numerous results.*

**Response:** The usage of “trend” and “dose-dependent difference” has been deleted. In addition, the lack of statistical analysis has been added as a limitation in Line 362.

*5. Strictly speaking, hypocalcemia is not an ADR, all the more since the calcimimetic-induced decrease of serum PTH goes along with a decrease in serum calcium, at least in ESKD patients on dialysis therapy. It would be interesting to have information on usual hypocalcemia-associated clinical symptoms and signs.*

**Response:** Although we understand the review comment, this was an *ad hoc* analysis of previously fixed data obtained in the head-to-head comparison study. As the reviewer pointed out, because of the clinical interest and significance of this study, future studies are needed to investigate the typical hypocalcemia-associated clinical symptoms and signs.

*6. In a related post-hoc analysis of the EVOLVE trial in hemodialysis patients receiving cinacalcet therapy - not quoted by the authors - Floege et al found that in the majority of hypocalcemia episodes changes in calcium administration were the preferred treatment option (DOI: 10.1016/j.kint.2017.12.014). In that trial, adjustments of cinacalcet or vitamin D sterol doses were made only in a minority of hypocalcemia episodes. This observation, together with the observation in the present study that high-dose VDRA treatment was associated with significantly higher serum FGF23 levels than low-dose VDRA treatment, and the notion that higher FGF23 levels are associated with higher mortality risk, would rather plead against systematic VDRA administration in dialysis patients on calcimimetic therapy. The authors may want to make this clear in the Discussion.*

**Response:** Accordingly, the discussion has been changed in Line 353, and the post-hoc analysis of the EVOLVE trial by Floege et al. has been added to the References section.

Minor remarks

*Abstract should tell that evocalcet was administered orally. Moreover, authors need to indicate, both in Abstract and in Methods section, that the head-to-head comparison of the parent trial was between evocalcet and cinacalcet.*

**Response:** The Abstract has been corrected by adding “oral” in Line 26. The Abstract and Materials and Methods sections have been corrected to clearly state that the previous phase 3 head-to-head comparison study investigated evocalcet and cinacalcet in Lines 24 and 89.

*Abstract L37. I wonder whether the term « clinical benefits » is appropriate here, at least as far as FGF23 is concerned. As to the lesser incidence of hypocalcemia I would like to know whether concomitant vitamin D receptor activator treatment was more effective in avoiding hypocalcemia-associated symptoms and signs.*

**Response:** “Clinical benefits” has been changed to “benefits” in Lines 39 and 374. All patients in the *ad hoc* analysis were treated with evocalcet and, therefore, further studies are needed to answer the reviewer’s comment.

*Introduction. The statement that IV administration of VDRA « is the standard therapy for patients with SHPT » is incorrect as such. At least in the Western world VDRA can by given either orally or intravenously for the treatment of SHPT.*

**Response:** Thank you for pointing this out; however, unlike Western countries, IV VDRA is the standard therapy for patients with SHPT and oral VDRA is used to supplement VD and/or treat hypocalcemia in Japan. To clarify the different application of oral VDRA, “in Japan” has been added in Line 57.

*Discussion, lines 238-284. The statement is inappropriate that « intact PTH levels were slightly lower in the LDG and HDG compared with the NDG » since the difference was not statistically significant.*

**Response:** The sentence “In this study, we found that over the 30 weeks of treatment, intact PTH levels were slightly lower in the LDG and HDG compared with the NDG” has been corrected to “Although it was not statistically significant, we found that over the 30 weeks of treatment, intact PTH levels tended to be slightly lower in the LDG and HDG compared with the NDG” in Line 303 to emphasize that it was not statistically significant.

**Reviewer #2:**

*This study has demonstrated that the association of oral evocalcet with vitamin D activator was beneficial, with lower synthesis of FGF-23, lower incidence of hypocalcemia and with more patients achieving the calcium target. This oral formulation of evocalcet, was also associated with a reduction of gastric symptoms when compared with oral cinacalcet.*

*This study confirms the benefit of the treatment of hyperparathyroidism with an association of a calcimimetic + vitamin D.*

*The reduction of gastric symptoms verified with evocalcet will contribute for a better adherence of this new oral treatment for hyperparathyroidism.*

*However, the intravenous administration of etelcalcetide after hemodialysis, assures the patient adherence to this kind of treatment.*

*Evocalcet will be very useful for the treatment of patients with hyperparathyroidism who develop gastric symptoms associated with cinacalcet and who have no conditions for a three times/week intravenous treatment. This will be the case, for instance, of DP patients.*

**Response:** We appreciate the reviewer’s comments on etelcalcetide and its comparison with evocalcet regarding its adherence and applications. Both drugs have advantages and disadvantages that can be used to provide the best available treatment according to each patient’s clinical characteristics and needs. In the previous head-to-head comparison study, the efficacy and safety of oral cinacalcet and evocalcet were compared and, therefore, we would like to keep the discussions on the comparisons between these two oral calcimimetics in this study.

**Reviewer #3:** *This paper reported an ad-hoc analysis results using patients enrolled in one treatment arm of a completed phase III trial. Patients were categorized into three groups according to average weekly VDRA dose. Many clinical outcomes and adverse drug reactions were compared among these three groups. However, almost all statistical analysis only employed basic statistical tests and multiple testing adjustment was not implemented. In addition, most statements about the dose effect of VDRA were without rigorous statistical evidences presented. A professional statistician is recommended to be involved for rigorous data analysis and drawing conclusions.*

*Specifically,*

*1. Figure 1 displayed longitudinal mean measurements for each group of patients. It would be helpful to also include variations in the plots. In addition, linear mixed models are recommended to do a formal comparison among three patients groups and make conclusions rather than just looking at the plots and stating there was a tendency of VDRA dose-dependent trend.*

**Response:** Standard deviations have been summarized in S2 Table. We appreciate the reviewer’s comment regarding the statistical analysis. The original phase 3 study, as well as this *ad hoc* analysis, was designed to statistically test the guideline target achievement rate among the treatment groups for mineral and bone disorder parameters, but not to test significant longitudinal trends in the repeated measures within each group or those among the groups. We are aware that a linear mixed model is a useful statistical tool whereby 30 weeks of measurements can be tested among the three groups unlike ANOVA. We will consider the reviewer’s comment when we design future studies. Please see our response to reviewer 1 comment 4 regarding the lack of statistical analyses on the VDRA dose-dependent trends.

*2. Table 1 implies that these three groups of patients were not balanced in all baseline measurements. Therefore, the corresponding result comparisons should consider adjusting for these possible confounding factors.*

**Response:** As the reviewer pointed out, we considered adjusting the outcome data with confounding factors, such as age, and baseline FGF23 and BAP levels. However, because of the small sample size (n=45 in the LDG group) it was impossible to adjust for such factors. Therefore, we have stated the potential for bias in Line 353.

*3. A conclusion from Figure 4 says "The slope of the FGF23 intact and C-terminal relationship increased after treatment with evocalcet at Week 30 compared with Week 0 in all patients, as well as by VDRA DG." First, the estimated slope were not reported. Second, a formal statistical analysis should be implemented here to provide support of such statement.*

**Response:** The estimated slopes for Week 0 and Week 30 in all patients, and those in three DGs, have been added to Fig. 4 (now changed to Fig. 5 after revision). The statistical analysis plan was created and approved for this study and, therefore, it is difficult to run an additional statistical analysis. Furthermore, because of the small sample size, we planned to find and discuss any *tendency* rather than statistical significances in this study.

Minor comments:

*1. The title of Table 2 says "Mean numbers and percentages...". However, outcomes in Table 2 are all binary ones. It is confusing why there would be mean numbers and percentages.*

**Response:** “Mean” has been deleted.

*2. It is also helpful to add in variation of those ratios to Figure 3.*

**Response:** Error bars have been added to Fig. 3 (now changed to Fig. 4 after revision).
