# Supplementary material for: Evocalcet with vitamin D receptor activator treatment for secondary hyperparathyroidism
Source: PLoS One. 2022 Feb 17;17(2):e0262829. doi: 10.1371/journal.pone.0262829 (PMC8853539; doi:10.1371/journal.pone.0262829)
Supplement: Attachment — Submitted filename: Response to Reviewers comments.doc [file pone.0262829.s005.doc]

Below you will find our point-by-point responses to all the reviewers’ comments.

**Reviewer #3:** *Previous comments related to rigorous statistical analysis were not fully addressed and hence this manuscript still does not reach the standard for publication. Authors are expected to fully address previous statistical comments again.*

**Response:** We have addressed all previous statistical comments. Please see our responses below.

*This is a post ad hoc analysis, so it is not convincible that no additional statistical analysis could be added because of a previously approved statistical analysis plan which is not submitted with this manuscript at all. There is one group of patients having relatively low number of patients (N=45), however, this sample size is not too small to adjust for any covariate. In addition, for a rigorous inference following any statistical analysis, 95% CI should be reported along with point estimate all the time. For example, the estimated slopes reported in Figure 5 should be reported along with the corresponding 95% CI.*

**Response:** Please see our response to the third comment below.

*Previous statistical comments*

*1. Figure 1 displayed longitudinal mean measurements for each group of patients. It would be helpful to also include variations in the plots. In addition, linear mixed models are recommended to do a formal comparison among three patients groups and make conclusions rather than just looking at the plots and stating there was a tendency of VDRA dose-dependent trend.*

**Response:** Differences in changes in intact PTH, and corrected calcium and phosphorus from baseline to Week 30 among the three treatment groups were analyzed with a repeated linear mixed model, as suggested. As you will find in the Results section on pages 12–13, significant differences in the intact PTH, cCa, and phosphorus levels were found between certain groups. However, no significant difference was observed for the intact PTH, cCa, or phosphorus level among the three dose groups when the levels were adjusted according to their respective baseline levels (all cases p > 0.95). These results therefore suggest that evocalcet exerts its effect regardless of VDRA use or its dose.

*2. Table 1 implies that these three groups of patients were not balanced in all baseline measurements. Therefore, the corresponding result comparisons should consider adjusting for these possible confounding factors.*

**Response:** We have added the between-group differences of the target parameter achievement rates adjusted for age (< 65 and ≥ 65 years), sex, and intact PTH level (< 500 pg/mL and ≥ 500 pg/mL) at baseline and previous cinacalcet treatment to Table 2.

*3. A conclusion from Figure 4 says "The slope of the FGF23 intact and C-terminal relationship increased after treatment with evocalcet at Week 30 compared with Week 0 in all patients, as well as by VDRA DG." First, the estimated slope were not reported. Second, a formal statistical analysis should be implemented here to provide support of such statement.*

**Response:** As suggested by the reviewer, changes in the ratio of intact-to-C-terminal FGF23 between Week 0 and Week 30 were analyzed with a generalized linear mixed model. As shown below, the slope of the FGF23 intact and C-terminal relationship increased after treatment with evocalcet at Week 30 compared with Week 0 in all patients (p = 0.0002) and patients in the NDG (p = 0.0005). These results suggest a reduction in FGF23 synthesis. Although no statistical significance was observed, the slopes tended to increase in the LDG (p = 0.0696) and HDG (p = 0.0741). Results of the statistical analysis have been included in the Results section on page 19.
